# Supplementary material for: Graphene controlled Brewster angle device for ultra broadband terahertz modulation
Source: Nat Commun. 2018 Nov 21;9:4909. doi: 10.1038/s41467-018-07367-8 (PMC6249283; doi:10.1038/s41467-018-07367-8)
Supplement: Supplementary file 1 — Supplementary Information [file 41467_2018_7367_MOESM1_ESM.pdf]

Graphene Controlled Brewster Angle Device for Ultra Broadband Terahertz Modulation

Zefeng Chen et al.

Supporting Information

Graphene Controlled Brewster Angle Device for Ultra Broadband Terahertz Modulation

Zefeng Chen[1,#], Xuequan Chen[1,#], Li Tao[1], Kun Chen[1], Mingzhu Long[1], Xudong Liu[1], Keyou Yan[1], Rayko I. Stantchev[1], Emma Pickwell-MacPherson[1, 2,]*, and Jian-Bin Xu[1,]*

[1]Electronic Engineering, The Chinese University of Hong Kong, Hong Kong, Shatin, NT, Hong Kong SAR, China

[2]Physics Department, Warwick University, Coventry, UK

# These authors contributed equally to this work.

* To whom correspondence should be addressed. Email: jbxu@ee.cuhk.edu.hk and e.pickwell.97@cantab.net

Supplementary Figures

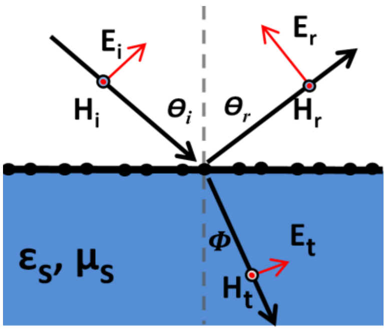

Supplementary Figure 1 Optical path diagram of incident light from air to media

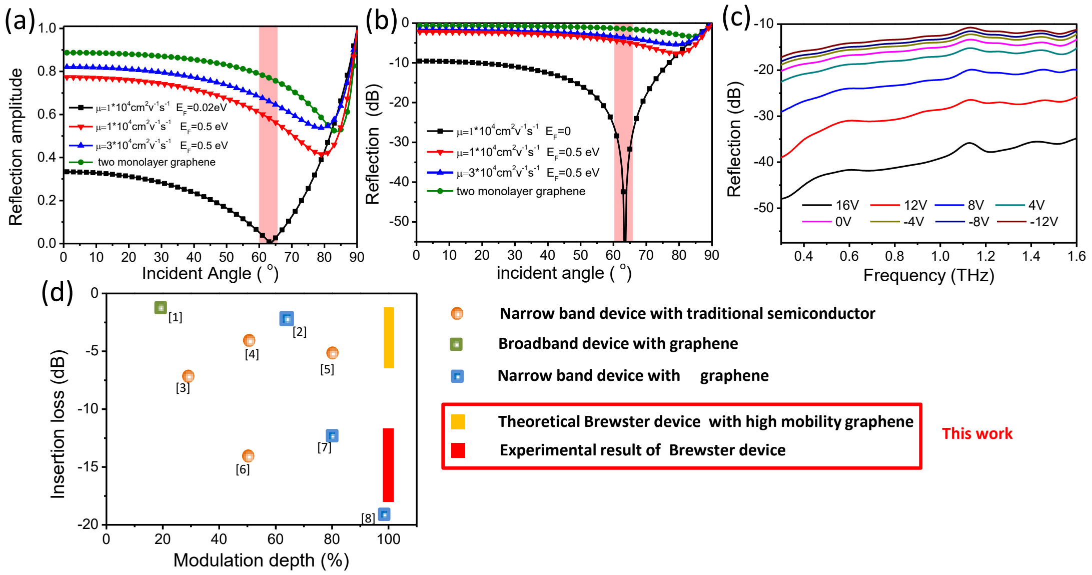

Supplementary Figure 2 (a) and (b) theoretical reflectance of Brewster device with high mobility graphene and the correspond reflection in dB scale (c) the experimental reflection (normalized to incident THz signal) in dB scale (d) summary of solid-state electrically driven THz modulators in terms of modulation depth versus insertion loss.

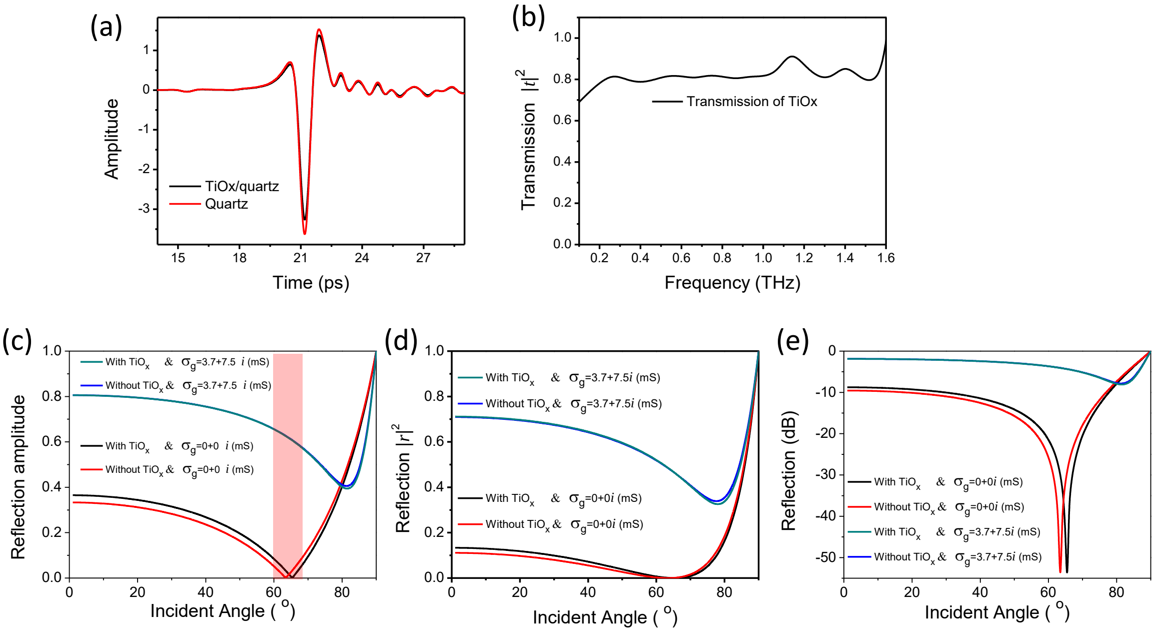

Supplementary Figure 3 Transmission of TiOx on Quartz (a) time domain spectrum (b) transmission spectrum in frequency domain. (c) Calculation results of reflection for Brewster device with and without TiOx (d) reflection in team term of power (e) reflection in dB scale

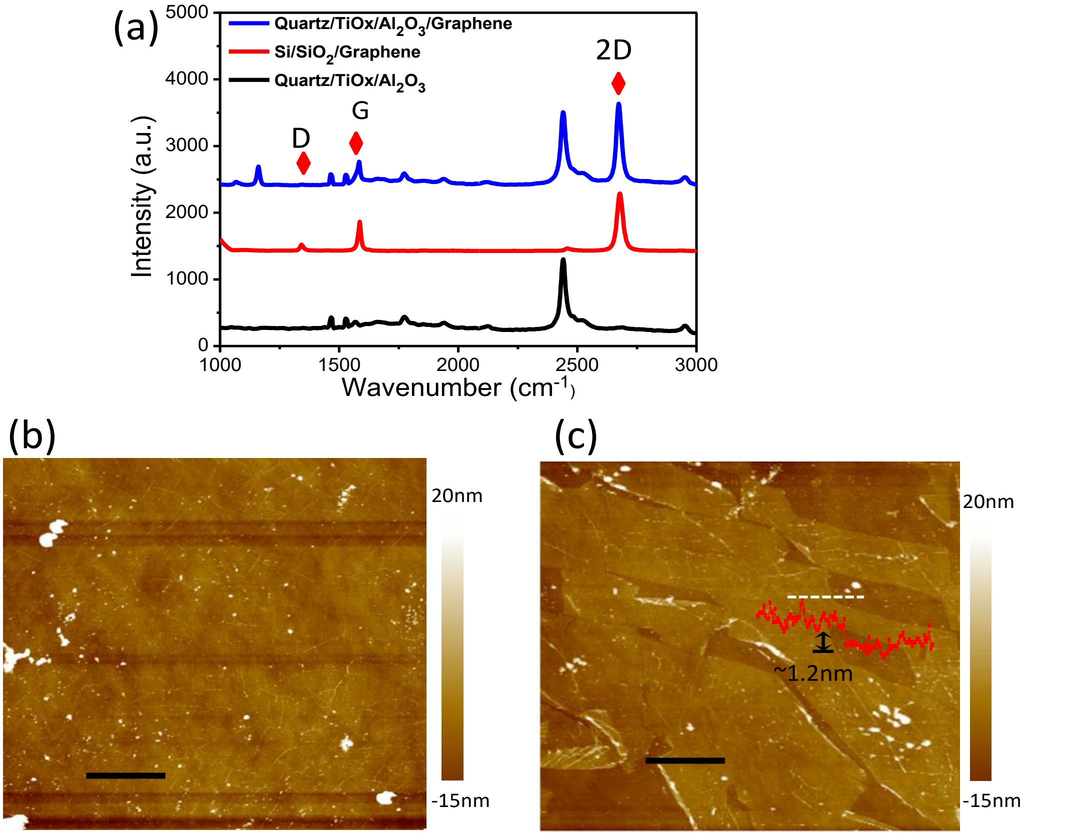

Supplementary Figure 4 (a) Raman spectrum of quartz/TiO_x/Al_2O_3 and graphene on

quartz/TiO_x/Al_2O_3 substrate and Si/SiO2 wafer. (b) and (c) tomography of graphene. The scale bar in (b) and (c) is 5 μm

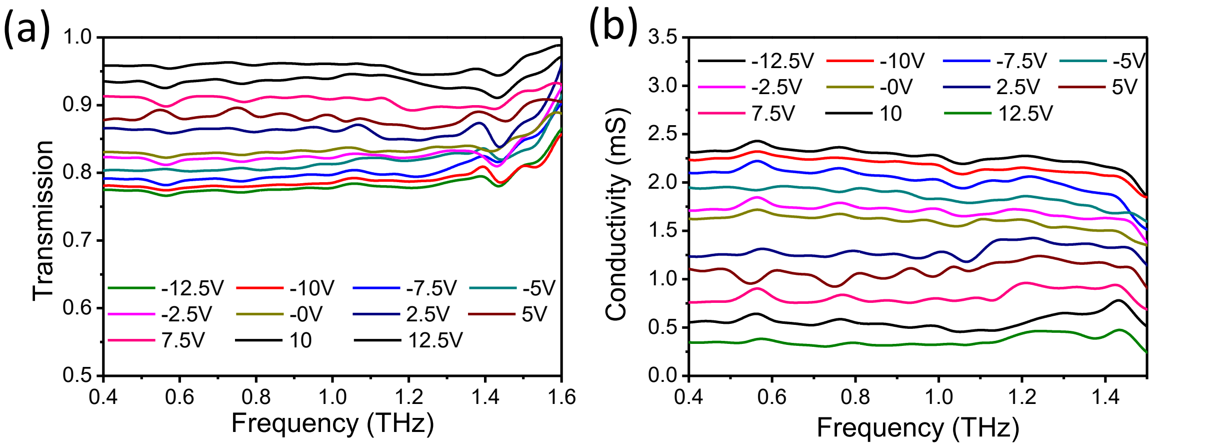

Supplementary Figure 5 (a) and (b) transmission spectra and conductivity of graphene under different gate voltages

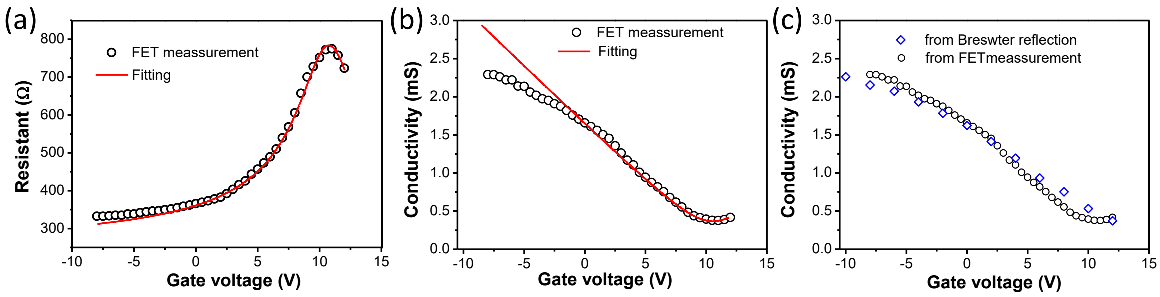

Supplementary Figure 6 (a) Transfer curve of graphene FET and the fitting curve (b) of conductivity extracted from FET meassurement and the fitted FET model. (c) Conductivity extracted from the FET meassurement and from the Brewster angle.

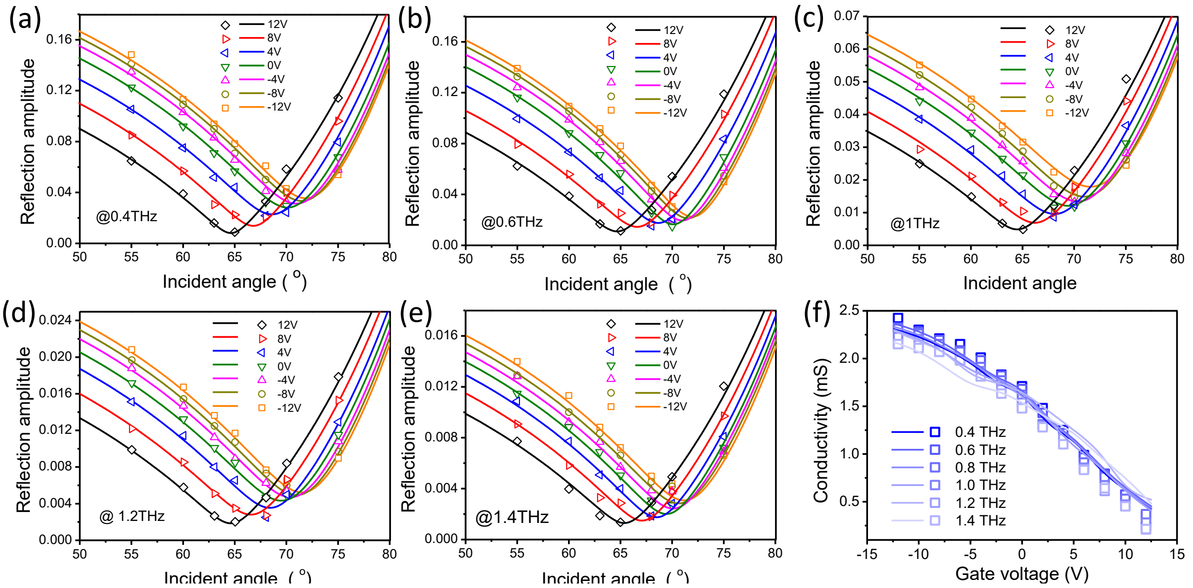

Supplementary Figure 7 (a) to (e) Experimental amplitude as a function of incident angle for the frequencies of 0.4 THz, 0.6 THz, 1 THz, 1.2 THz and 1.4 THz. Open symbols present the experimental amplitude as a function of incident angle at different gate voltages. Solid lines are fitting results. (f) conductivity as function of gate voltage. Open squares are the fitted conductivity from the Brewster reflection; lines are the conductivity extracted from transmission spectra.

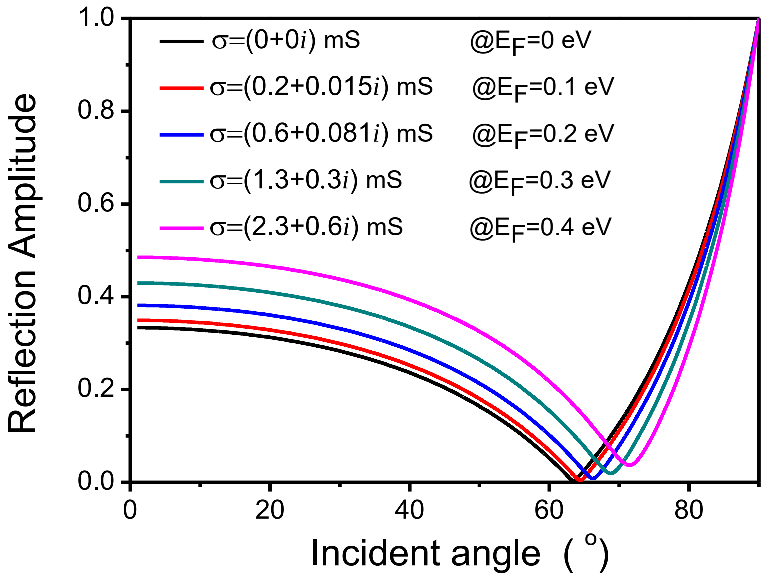

Supplementary Figure 8 Theoretical result of tunable Brewster angle when $\mu = 1.3 \times 10^3 \mathrm{cm^2V^{-1}s^{-1}}$, the exact mobility of graphene sample in the experiment.

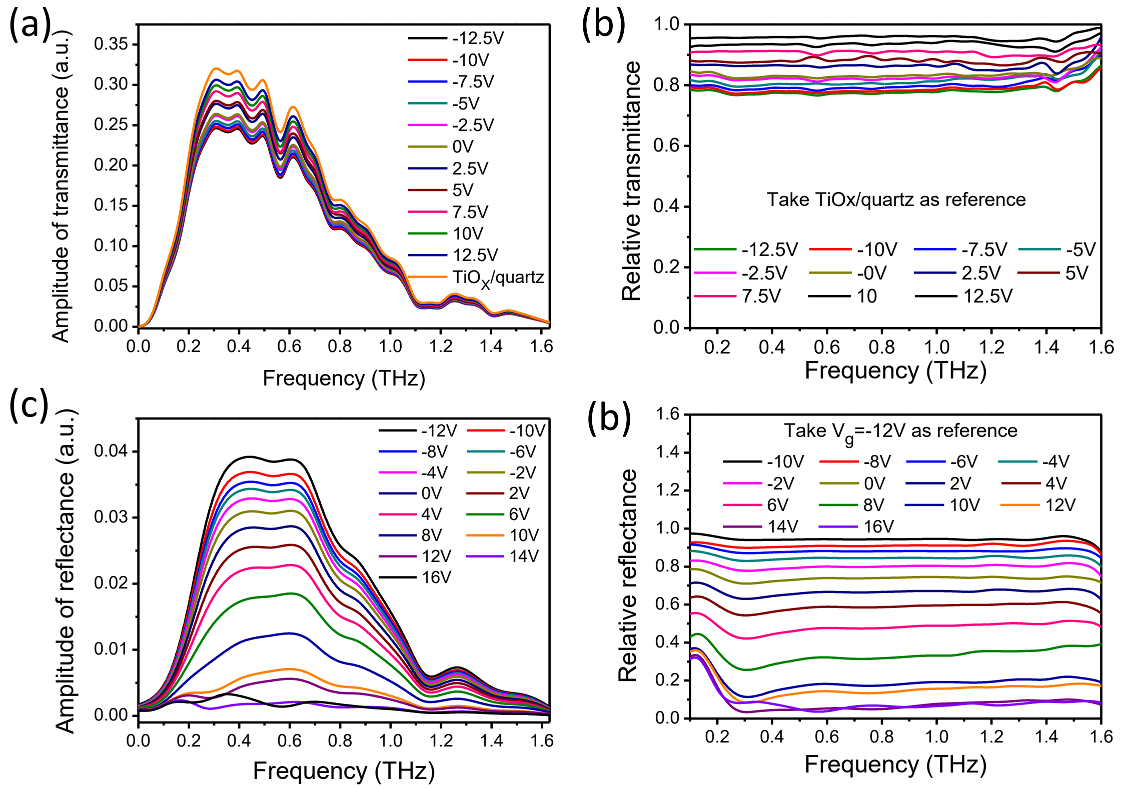

Supplementary Figure 9 (a) transmission amplitude extracted from THz time dominate pulse through FFT. (b) relative transmittance. (c) reflection amplitude extracted from THz time dominate pulse through FFT. (d) relative transmittance.

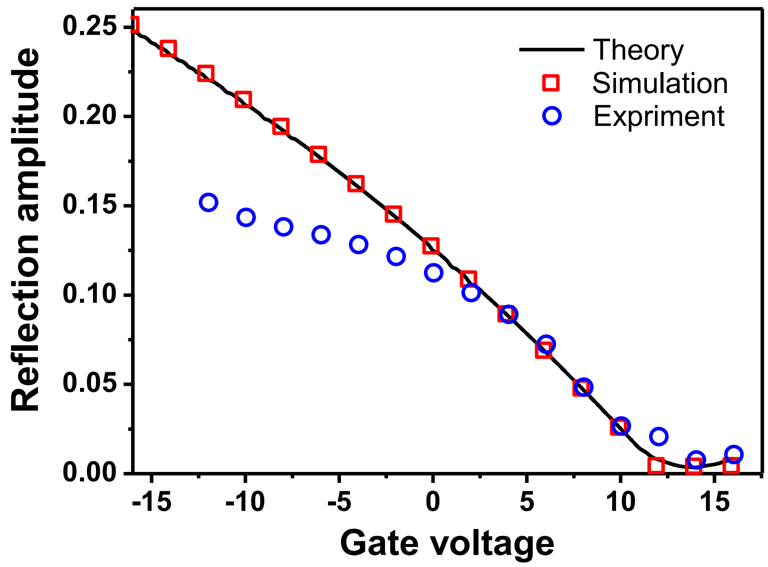

Supplementary Figure 10 Simulated (red open squares), theoretical (black curve) and experimental (blue open circles) values of intensity modulation.

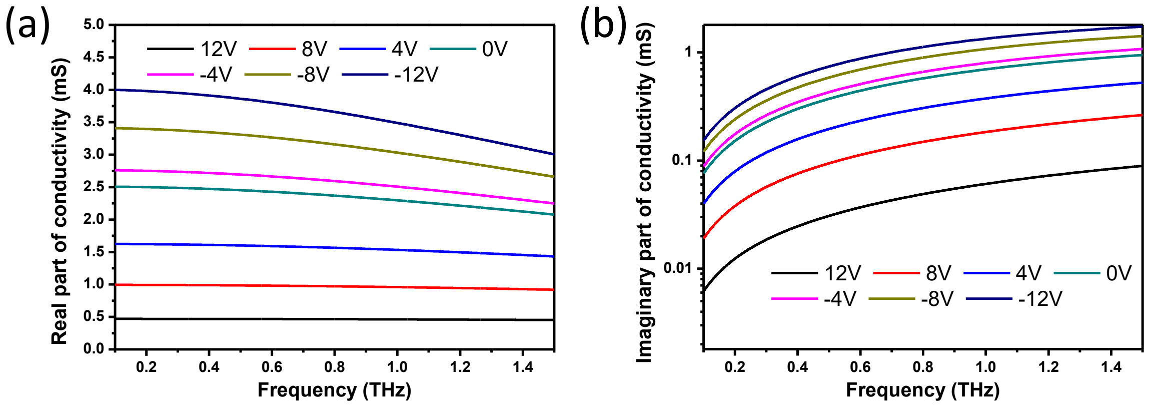

Supplementary Figure 11 (a) and (b) the real and imaginary part of the conductivity of graphene as a function of frequency

Supplementary Notes

Supplementary Note 1. Fresnel Function for Graphene/Quartz

Consider a system， as shown in Supplementary Figure 1, graphene with conductivity σ_g and media with relative permittivity ε_s, and relative permeability μ_s. A beam of *p*-polarization light is incident from air to graphene. The incident light will be separated into three parts, namely the reflection from graphene, the transmission into the medium, and the absorption by graphene.

According to Maxwell equations, the boundary conditions of the electric field and magnetic field are:

$$E_i\cos\Theta - E_r\cos\Theta = E_t\cos\Phi, \qquad \text{[Supplementary Equation 1]}$$

$$H_i + (H_r - H_t) = \sigma_g E_t\cos\theta_r, \qquad \text{[Supplementary Equation 2]}$$

where $E_i(H_i)$, $E_r(H_r)$ and $E_t(H_t)$ is the electric (magnetic) field of incident, reflected and transmitted light, respectively. On the other side, according to Snell's law, we can obtain the relation between the incident angle θ_i, reflection angle θ_r, and refraction angel Φ. That is $\theta_i = \theta_r$ and $\sqrt{\mu_s\varepsilon_s}\sin\theta_i = \sqrt{\mu_0\varepsilon_0}\sin\Phi$, where $\sqrt{\mu_0\varepsilon_0} = 1$ $n = \sqrt{\mu_s\varepsilon_s}$ and the permittivity and permeability of air is ε_0, μ_0.

By combining Supplementary Equations 1 and 2, we can determine the reflection and transmission coefficients for *p*-polarization light:

$$r_{\mathrm{p}} = \frac{\sqrt{\varepsilon_{\mathrm{s}}\mu_{\mathrm{s}}}\cos\theta_{\mathrm{i}} - \sqrt{\varepsilon_0\mu_0}\cos\Phi + Z_0\sigma_{\mathrm{g}}\cos\theta_{\mathrm{i}}\cos\Phi}{\sqrt{\varepsilon_{\mathrm{s}}\mu_{\mathrm{s}}}\cos\theta_{\mathrm{i}} + \sqrt{\varepsilon_0\mu_0}\cos\Phi + Z_0\sigma_{\mathrm{g}}cos\theta_{\mathrm{i}}\cos\Phi}$$ [Supplementary Equation 3]

Supplementary Note 2. Insertion loss

At a fixed incident angle, the graphene controlled Brewster angle device can work as intensity modulator. The insertion loss of this device is $IL = 20\log_{10}(r_{\mathrm{max}})$. r_{max} is proportional to the conductivity of graphene, which is related to graphene's mobility μ and Fermi energy E_F. Recently, the highest mobility of 80000 $cm^2\ V^{-1}s^{-1}$ is demonstrated in CVD growth graphene in room temperature [9,11]. Therefore, in the calculation we take 10000 $cm^2V^{-1}s^{-1}$ and 30000 $cm^2V^{-1}s^{-1}$ as the mobility of graphene. r_{max} is about 0.5 and 0.7 respectively, corresponding to insertion loss of -4.5dB and -3dB, shown in Supplementary Figure 2(a) and (b). In fact, high conductivity can be achieved by using two-monolayer graphene, where a dielectric material is sandwiched by two-monolayer graphene. In this case, r_{max} can be up to 0.8 and insertion loss is -2dB. In our experiment, commercial graphene with mobility around 1300 $cm^2\ V^{-1}s^{-1}$ (shown in Supplementary Note 6) is used in the device. As a result, the maximum reflectance is much lower than that of the theoretical results. In this case, the insertion loss is about -11dB to -15dB, as shown in Supplementary Figure 2 (c).

Supplementary Figure 2 (d) shows the summary of solid-state electrically driven THz modulators in terms of modulation depth versus insertion loss. Narrow band device (electromagnetic resonance structure with graphene or traditional semiconductor) can obtain high modulation depth but together with high loss. The modulator depth of graphene-controlled Brewster device is better than other kind of device and the experimental loss is comparable with some narrow band device[5,7,8]. Through fabrication and material optimization, it is possible to reduce the loss of the Brewster device to the theoretical value and achieve excellent performance.

Supplementary Note 3. Characterization of TiOx

The THz signals for quartz and quartz/TiOx are shown in Supplementary Figure 3 (a), which show the same pulse shape. The corresponding transmission is about 0.8 over the frequency of 0.2 THz to 1.6 THz, Supplementary Figure 3 (b). According to Fresnel equation, the transmission of a free standing ultra-thin conducting film in air is $T = |t|^2 = \left|\frac{2}{2+Z_0\sigma}\right|^2$, where σ=0.5 mS is the conductivity of TiOx and $Z_0 = 377\ \Omega$ is the impedance of air. So we

can estimate the transmission of TiOx is about 0.8, which corroborates well with the experimental result.

To clear the influence of TiOx on our device, we calculate the reflectance of Brewster device with and without TiOx. In the calculation, the conductivity of TiOx is set to be 0.5 mS; the conductivity of graphene is set to be from 0 mS to (3.7+7.5i) mS, which corresponds to the Fermi energy from 0 eV to 0.4 eV. Supplementary Figure 3(c)is the reflection amplitude for the device with and without TiOx when the graphene conductivity is at 0 mS and (3.7 +7.5i) mS. When the conductivity of graphene is zero, the Brewster angle (where the reflection amplitude is minimum) for the device with TiOx is shifted from 63° to 65° due to the conductivity of TiOx. This indicates that when using this device with TiOx for an intensity modulator, the incident angle should be 65°.

When the conductivity of graphene increases to (3.7 +7.5i) mS, the Brewster angle for the two devices are almost the same and the minimum reflection amplitude for the device with TiOx is little smaller than that without TiOx. More importantly, at the incident angle of 65°, the reflection amplitude for the device with and without TiOx is almost the same. In the term of power $|r|^2$, they are also almost the same (Supplementary Figure 3(d)). This means that the TiOx of 0.5 mS do not influence the modulation depth. Supplementary Figure 3(e) are the reflection in dB scale, where the reflection for $\sigma_{\mathrm{g}} = (3.7 + 7.5\mathrm{i})\ \mathrm{mS}$ is corresponding to the insertion loss of the device. We can see that the device with TiOx does not suffer significantly increased loss (or very little loss) compared to the device without TiOx. The loss due to a conducting layer (TiOx and graphene, here) is proportional to the conductivity. $L_{\mathrm{insertion}} \infty \sigma \cdot E^2$, where σ is the total conductivity. On the other hand, when the conductivity increases, the conducting layer surface reflects more THz light and less E-field penetrates into the conducting layer, which decreases the E term in the above equation. As a result, introducing the TiOx layer does not cause palpable loss in the device.

In short, the TiOx layer does introduce 20% of power (about -1dB) to be absorbed/reflected when THz wave passes through the TiOx layer. However, in our Brewster device, the TiOx layer does not introduce palpable influence on both modulation depth and loss.

Supplementary Note 4. Raman and AFM characterization of graphene

The Raman spectra are shown in Supplementary Figure 4 (a). when the graphene is transferred on to Si/SiO_2 wafer, there are three characteristic peaks: D, G and 2D peaks. When graphene is transferred to our prepared substrate quartz/TiO_x/Al_2O_3, we can not only observe graphene's three characteristic peaks, but some other peaks contributed by the substrate (the black line). For graphene on the substrate quartz/TiO_x/Al_2O_3, the intensity of D peak is very low and the intensity of 2D is two times over that of G. These indicate graphene is single layer with low defect. The tomography of graphene (Supplementary Figure 4(b)) shows that the surface of graphene very flat, except same folds due the stress during the transfer process. To characterize the thickness of graphene, we measure tomography of graphene at the edge of the film, where graphene fragment can be found (Supplementary Figure 4 (c)). The thickness of graphene is about 1.2 nm, which is thick than the intrinsic thickness. This is because of some fine PMMA residue and contaminants on the graphene surface after transfer process increase the thickness.

Supplementary Note 5. Transmission Spectra of Graphene

To obtain the conductivity of the graphene, we measured the time-domain THz pulse transmitted through both the device (graphene/Al_2O_3/TiO_x/quartz) and the reference sample (quartz). The transmission spectra (Supplementary Figure 5(a)) in the frequency-domain are determined by Fourier transform of the time-domain data. By applying the standard thin-film approximation [12, 13], and knowing that the relationship between transmission spectra and the frequency-dependent conductivity is:

$$t = \frac{E}{E_0} = \frac{\sqrt{\varepsilon_s \mu_s} + \sqrt{\varepsilon_0 \mu_0}}{\sqrt{\varepsilon_s \mu_s} + \sqrt{\varepsilon_0 \mu_0} + Z_0 \sigma_g} \qquad \text{[Supplementary Equation 4]}$$

where E and E_0 are the transmitted E-fields from the graphene-substrate sample and bare substrate. We can deduce that the conductivity of graphene $\sigma_{\mathrm{g}} = \frac{(\sqrt{\varepsilon_{\mathrm{s}}\mu_{\mathrm{s}}}+\sqrt{\varepsilon_0\mu_0})(1-t)}{Z_0 r}$, which is shown in Supplementary Figure 5(b).

Supplementary Note 6. Graphene Field-Effect Transistor (G-FET)

The transfer curve of graphene FET is depicted in Supplementary Figure 6(a). The charge neutrality point V_{Dirac} is found at around 12V. Before this point, holes dominate the conductivity of graphene and the conductivity increases as the magnitude of gate voltage increases. The hole mobility and density can be obtained by fitting the following equations:

$$R_{\mathrm{total}} = R_{\mathrm{contact}} + R_{\mathrm{channel}} = R_{\mathrm{contact}} + \frac{L/W}{ne\mu} \qquad \text{[Supplementary Equation 5]}$$

$$n = \sqrt{n_0^2 + n_{\mathrm{g}}^2} \qquad \text{[Supplementary Equation 6]}$$

$$n_{\mathrm{g}} = C_0(V_{\mathrm{g}} - V_{\mathrm{Dirac}})/e \qquad \text{[Supplementary Equation 7]}$$

$$\sigma = \frac{L}{W}/(R_{\mathrm{total}} - R_{\mathrm{contact}}) \qquad \text{[Supplementary Equation 8]}$$

where R_{total} represents the total resistance of the device composed of contact (R_{contact}) and channel resistance (R_{channel}), e is the elementary charge, μ and n are the holes mobility and density in graphene, respectively, and W and L are the width and length of the channels. $n_0$is the residual carrier density originating from charge impurities and n_{g} is the carrier density contributed from the back-gate bias. C_0 ~140 nF cm^{-2} is the areal capacitance of 50 nm-thick Al_2O_3 dielectric[14]. The extracted residual carrier density is ~0.87×10^{12} cm^{-2}, and the mobility is about ~1.3×10^{3} cm^{2}V^{-1}s^{-1}).

It is worth to mention that the fitting result in the positive gate region matches well with experimental data, while in the negative region the conductivity is higher than experimental values, as shown in Supplementary Figure 6(b). In this region, conductivity of graphene starts to be saturated because of the scattering effect of high density carriers, which lowers the conductivity of graphene.

Supplementary Note 7. Fitted Brewster Angle and Conductivity for Different Frequencies

The amplitude as a function of incident angle for the frequency of 0.4 THz, 0.6 THz, 1 THz, 1.2 THz and 1.4 THz is shown in Supplementary Figure 7(a)-(e). The conductivity of graphene is extracted by fitting the experimental data to Eq. (1). The fitted graphene conductivity is shown in Supplementary Figure 7(f). For these frequencies, the fitted conductivity is consistent with the conductivity extracted from the device's THz transmission spectra.

Supplementary Note 8. Graphene-controlled Brewster angle

The exact value of graphene's parameter is achieved when fitting the measurement results to the Brewster reflection model, also can be extracted from the transmission spectrum, as well as field effect transistor (FET). The mobility is about $1.3 \times 10^3 \mathrm{cm}^2\mathrm{V}^{-1}\mathrm{s}^{-1}$ and the conductivity ranging from about 0.4 mS to 2.4 mS. With this parameter, the theoretical results is achieved through the physical model, as shown in Supplementary Figure 8.

Supplementary Note 9. Amplitude extracted from THz time dominate pulse

The transmission amplitude is shown in Supplementary Figure 9(a), where maximum THz radiation is at 0.3 THz to 0.6 THz, and gradually decay as the frequency increase. The corresponding relative transmittance (defined as $\frac{E_\mathrm{t}}{E_\mathrm{Ref}}$, E_Ref is the THz amplitude of TiO_x/quartz) is shown in Supplementary Figure 9(b). Although, the THz radiation in high frequency region (>1THz) signal is weak (Supplementary Figure 9(a)), tunable relative transmittance of about 20% is observed. (Supplementary Figure 9 (b)).

The reflection amplitude in Brewster configuration with incident angle of 65 °is shown in Supplementary Figure 9(c). The distribution of THz radiation amplitude in frequency domain is similar to that of transmission signal. Although, the THz radiation in high frequency region (>1THz) signal is weak, the modulation effect of relative reflectance (defined as $\frac{E_\mathrm{r}}{E_\mathrm{Ref}}$, E_Ref

is the reflection amplitude at the gate voltage of -12V) is still excellent, both spectrally flat and highly tunable, as shown in Supplementary Figure 9 (d). This reflects the excellent performance of the graphene-controlled Brewster angle device.

Supplementary Note 10. Simulated, Theoretical and Experimental Intensity Modulation

With the conductivity given by Supplementary Equation 8, we calculated the reflection amplitude according to the theory (Supplementary Equation 3), as show in Supplementary Figure 10. To confirm this theoretical result, we also used commercial software COMSOL to conduct the simulation. The simulated results match very well with the theory. The experimental result at the positive gate region also agrees well with the simulation and theory. However, as the gate voltage decreases, the experimental reflection amplitude becomes smaller than that from the theory and simulation. The reason is that the practical conductivity of graphene in this gate voltage region is slightly smaller than that of the FET model, as shown in Supplementary Figure 6(b).

Supplementary Note 11. Graphene's Conductivity under Dude Model

In the THz region, the conductivity of graphene can be described by the Drude model, which is $\sigma_{\mathrm{g}} = \frac{\mathrm{i}D}{\pi(\omega+\mathrm{i}\Gamma)}$, where ω is the frequency, Γ the scattering rate, and D is the Drude weight. For the carriers in graphene, $D = (v_{\mathrm{F}}e^2/\hbar)\sqrt{\pi n}$ and$\Gamma = ev_{\mathrm{F}}/(\hbar\mu\sqrt{\pi n})$, where $v_{\mathrm{F}} = 1 \times 10^6 \mathrm{ms}^{-1}$ is the Fermi velocity; and n and μ are the carrier concentration and carrier mobility of graphene, respectively. The carrier concentration is contributed from the residual carrier density n_0 and the gate effect, which can be described as $n = \sqrt{n_0^2 + n_{\mathrm{g}}^2}$. The values for $n_0 = 0.87 \times 10^{12}\mathrm{cm}^{-2}$ and $\mu = 1.3 \times 10^3 \mathrm{cm}^2\mathrm{V}^{-1}\mathrm{s}^{-1}$ are given by the fitting of result of G-FET in Supplementary Figure 6. With these in hand, we can achieve a dynamic conductivity model of graphene for our device, as follows:

$$\sigma = \frac{\mathrm{i}(v_{\mathrm{F}}e^2/\hbar)\sqrt{\pi n}}{\pi\left(\omega + \frac{\mathrm{i}ev_{\mathrm{F}}}{\hbar\mu\sqrt{\pi n}}\right)} \qquad \text{[Supplementary Equation 10]}$$

$$n = \sqrt{n_0^2 + (C_0(V_{\mathrm{g}} - V_{\mathrm{Dirac}})/e)^2} \qquad \text{[Supplementary Equation 11]}$$

The real part and imaginary part of conductivity as a function of frequency under

different gate voltage are shown in Supplementary Figure 11. The real part is weakly depended on the frequency, while the imaginary part, which comes from the plasmonic effect of graphene, increases as the frequency increases.

Supplementary References

[1] B. Sensale-Rodriguez, R. Yan, M. M. Kelly, T. Fang, K. Tahy, W. S. Hwang, D. Jena, L. Liu & H. G. Xing, ‘‘Broadband graphene terahertz modulators enabled by intraband transitions,’’ Nature Commun., 3, 780, (2012)

[2] B. Sensale-Rodriguez, R. Yan, S. Rafique, M. Zhu, W. Li, X. Liang, D. Gundlach, V. Protasenko, M. M. Kelly, D. Jena, L. Liu & H. G. Xing, Extraordinary control of terahertz beam reflectance in graphene electro-absorption modulators, Nano Lett., 9, 4518 (2012)

[3] H. T. Chen, J. F. O’Hara, A. K. Azad, A. J. Taylor, R. D. Averitt, D. B. Shrekenhamer & W. J. Padilla, Experimental demonstration of frequency-agile terahertz metamaterials, Nature Photon., 2, 295(2008)

[4] H. T. Chen, W. J. Padilla, J. M. O. Zide, A. C. Gossard, A. J. Taylor & R. D. Averitt, Active terahertz metamaterial devices, Nature, 444, 597–600, (2006)

[5] H. T. Chen, W. J. Padilla, M. J. Cich, A. K. Azad, R. D. Averitt & A. J. Taylor, A metamaterial solid-state terahertz phase modulator, Nature Photon., 3, 141 (2009)

[6] H. Chen, H. Lu, A. Azad, R. Averitt, A. Gossard, S. Trugman, J. O’Hara & A. Taylor, Electronic control of extraordinary terahertz transmission through subwavelength metal hole arrays, Opt. Exp., 16, 7641 (2008)

[7] S. H. Lee, M. Choi, T.-T. Kim, S. Lee, M. Liu, X. Yin, H. K. Choi, S. S. Lee, C.-G. Choi, S.-Y. Choi, X. Zhang & B. Min, Switching teraherz waves with gate-controlled active graphene metamaterials, Nature Mater., 11, 936 (2012)

[8] Osman Balci, Nurbek Kakenov, Ertugrul Karademir, Sinan Balci, Semih Cakmakyapan, Emre O. Polat, Humeyra Caglayan, Ekmel Özbay & Coskun Kocabas, Electrically switchable metadevices via graphene, Science Advances, 1, eaao1749(2018)

[9] Luca Banszerus, Michael Schmitz, Stephan Engels, Jan Dauber, Martin Oellers, Federica Haupt, Kenji Watanabe, Takashi Taniguchi, Bernd Beschoten & Christoph Stampfer,

Ultrahigh-mobility graphene devices from chemical vapor deposition on reusable copper, Science Advances, 6, e1500222(2015)

[10] D.A. Boyd, W.-H. Lin, C.-C. Hsu, M.L. Teague, C.-C. Chen, Y.-Y. Lo, W.-Y. Chan, W.-B. Su, T.-C. Cheng, C.-S. Chang, C.-I. Wu & N.-C. Yeh, Single-step deposition of high-mobility graphene at reduced temperatures, Nature Communications, 6, 6620 (2015)

[11] Soo Min Kim, Allen Hsu, Min Ho Park, Sang Hoon Chae, Seok Joon Yun, Joo Song Lee, Dae-Hyun Cho, Wenjing Fang, Changgu Lee, Tomás Palacios, Mildred Dresselhaus, Ki Kang Kim, Young Hee Lee & Jing Kong, Synthesis of large-area multilayer hexagonal boron nitride for high material performance, Nature Communications 6, 8662 (2015)

[12] M. Tinkham, Energy Gap Interpretation of Experiments on Infrared Transmission through Superconducting Films. Phys. Rev. **104**, 845 (1956)

[13] F. A. Hegmann, O. Ostroverkhova, D. G. Cooke, Probing Organic Semiconductors with Terahertz Pulses. Chapter 7, pp.367-425, in *Photophysics of Molecular Materials*, Wiley-VCH Verlag GmbH & Co. KGaA, Weinheim, Germany, 2006.

[14] Seyoung Kim, Junghyo Nah, Insun Jo, Davood Shahrjerdi, Luigi Colombo, Zhen Yao, Emanuel Tutuc & Sanjay K. Banerjee, Realization of a high mobility dual-gated graphene field-effect transistor with Al_2O_3 dielectric. Appl. Phys. Lett. **94**, 062107 (2009)
